# Compatibility of concurrent aerobic and resistance training on maximal aerobic capacity in sedentary males

**Published:** 2009-04

**Authors:** BS Shaw, I Shaw

**Affiliations:** Department of Sport, Rehabilitation and Dental Sciences, Tshwane University of Technology, Pretoria; Department of Marketing and Sport Management, Vaal University of Technology, Vanderbijlpark

## Abstract

**Summary:**

Aerobic and resistance training are often performed concurrently by inactive individuals and those patients undergoing cardiac rehabilitation, despite contradictory findings that this mode of training may impair the development of maximal aerobic capacity (VO_2max_). The aim of the study, therefore, was to compare the effects of 16 weeks of aerobic, resistance and concurrent aerobic or resistance training on VO_2max_ development.

Fifty apparently healthy males (25 years ± 8 months) were randomly assigned to a non-exercising control group (NonG) (*n* = 12), an aerobic training group (AerG) (*n* = 12), a resistance-training group (ResG) (*n* = 13), or a concurrent aerobic and resistance-training group (ConG) (*n* = 13). VO_2max_ was measured pre- and post-experimentally using a continuous on-line oxygen analyser. Aerobic training consisted of exercise using a combination of treadmills, rowers, steppers and cycle ergometers, whereas resistance training consisted of eight prescribed exercises performed for three sets of 15 repetitions at 60% of the estimated one-repetition maximum (1-RM). In an attempt to equalise exercise duration across all three experimental groups, concurrent aerobic and resistance training consisted of a combination of aerobic training at 60% of heart rate maximum, and resistance training for two sets of 15 repetitions at 60% of the estimated 1-RM.

The NonG were found to have decreased their VO_2max_ by 3.36%, whereas the ResG increased their mean VO_2max_ by 13.16%. The AerG and ConG increased their mean VO_2max_ by 34.12 and 29.58%, respectively.

In conclusion, concurrent training did not significantly interfere with development of aerobic capacity in sedentary males when compared to aerobic training. Therefore, this investigation did not support the concept of the universal nature of the interference effect that supposes the superiority of a single mode of training.

## Summary

A wealth of literature exists demonstrating the cardiovascular benefits of aerobic training. One such benefit of aerobic training is an increased cardio-respiratory endurance, which in turn reduces the relative risk of death associated with lack of maximal aerobic capacity (VO_2max_). This is because an increased VO_2max_ has concomitant cardio-protective effects, which include minimised left ventricular hypertrophy, increased levels of erythrocytes and haemoglobin and increased maximal capacity of the cardio-respiratory system.^1^

Compared with aerobic training, resistance training has produced conflicting results regarding its effect on VO_2max_,^1^-^4^ and despite resistance training sometimes increasing VO_2max_, it appears to be less effective than aerobic training.^5^-^9^ Even though resistance training is considered less effective, inactive individuals continue to use both aerobic and resistance training in an attempt to increase VO_2max_. This is in spite of research not convincingly demonstrating the effectiveness of this combined mode of training on VO_2max_.^2^,^4^,^10^-^17^

Nevertheless, it is hypothesised that the addition of resistance training to aerobic training could improve VO_2max_ in certain populations. This is since increases in strength and muscle mass (which improve mechanical efficiency, force–velocity relationships, blood lactate clearance and buffering mechanisms, and motor unit recruitment patterns) have been associated with increases in VO_2max_.^1^,^5^,^6^,^10^,^18^ Due to the above conflicting findings and scarcity of research on the ability of resistance and concurrent training to improve VO_2max_, the purpose of the study, therefore, was to compare the effects of 16 weeks of aerobic training, concurrent resistance and aerobic, or resistance training on VO_2max_ in previously untrained subjects.

## Methods

Fifty inactive men who volunteered to participate in this study (mean age: 25 years and six months), were selected after meeting the following final criteria for participation: being inactive, weight stable and on no dietary programme six months prior to the study, not using any pharmacological agent or dietary supplement known to affect VO_2max_, and free of medical conditions that could have prohibited exercise. Demographic data are presented in Table 1. Subjects were age-matched and randomly assigned using a random-numbers table to an aerobic training group (AerG) (*n* = 12), a resistance-training group (ResG) (*n* = 13), a concurrent aerobic and resistance-training group (ConG) (*n* = 13), or a non-exercising control group (NonG).

**Table 1:** Physical Characteristics Of Subjects

*Group*	*n*	*Age (years)*	*Height (cm)*	*Body mass (kg)*
AerG	12	25 ± 5.6	176.8 ± 3.8	74.7 ± 8.2
ResG	13	25 ± 3.5	175.5 ± 5.6	69.1 ± 8.5
ConG	13	26 ± 3.1	178.7 ± 7.0	85.0 ± 12.8
NonG	12	25 ± 2.4	179.3 ± 11.9	80.3 ± 12.8

Values are means ± standard deviation.

At a scheduled introductory session, the risks and procedures of the study were explained to the subjects. They were then shown the proper technique for each exercise utilised in their individualised training programmes. Prior to participation in the investigation, all volunteers gave written informed consent and underwent a medical screening history and physical examination, and they were allowed to discontinue the study at any time. This investigation was approved by the Rand Afrikaans University, Department of Human Movement Studies’ Research Committee.

All subjects underwent a similar battery of tests before and after the 16-week intervention period. All were evaluated in the post-absorptive state following a 12- to 14-hour fast and at least 48 hours prior to or following any exercise. Subjects were weighed in kilograms (to the nearest 0.1 kg) on a calibrated medical scale (Mettler DT Digitol, Mettler-Toledo AG, Ch-8606 GreiFensee, Switzerland) wearing only running shorts. Each subject’s stature was measured in centimetres (to the nearest 0.1 cm) via a standard wall-mounted stadiometer. Percentage body fat was calculated according to the equation of Jackson and Pollock (1978):^19^ Percentage fat = 100 (4.95/D_b_ – 4.5), where D_b_ (body density in g/cm^3^) = 1.120 – 0.00043499 (Σ7) + 0.00000056 (Σ7) – 0.00028826 (age), and Σ7 = sum of the seven skinfolds (in mm).

The triceps, subscapular, supra-iliac, abdominal, frontal thigh, mid-axilla and pectoral skinfolds were measured on the righthand side to the nearest 0.1 mm using a manual skinfold caliper (Harpenden John Bull, British Indicators Ltd., England).

VO_2max_ was measured directly at the start and completion of the experimental period using a continuous on-line oxygen analyser (OXYCON-5, Mijnhardt, Bunnik, Netherlands) and cycle ergometer (Monark Ergo Trainer 810, Monark, Vargberg, Sweden). Readings of oxygen consumption (VO_2_) were recorded every 30 seconds over the test period using the metabolic cart (OXYCON-5, Mijnhardt, Bunnik, Netherlands) and a Hans Rudolph two-way non-rebreathing valve (T-shape configuration model 2700B) (Wynadotte, Kansas City, United States of America). The VO_2max_ was determined at an altitude of 1 650 m above sea level and at a controlled temperature of 21 to 23° C.

The graded YMCA cycle ergometry protocol was utilised and if needed, extra stages of 25 watts were added to ensure that all subjects reached their respective maximums. The VO2max test was terminated when VO_2_ or heart rate decreased or remained unchanged in response to increases in workload, a respiratory quotient value of 1.14 and/or a rating of perceived exertion of 20 was reached, or the subjects experienced severe leg muscle fatigue or requested to stop. Workload was also considered maximal when successive increments in work intensity resulted in VO_2_ differences of not more than 1 ml of oxygen per kg per minute (ml O_2_.kg^-1^.min^-1^).

## Periodised training programmes

All subjects were under direct supervision during the training sessions and were familiarised with the equipment before commencement of the experimental programme. Exercise sessions were preceded by and concluded with five minutes of easy cycling at a heart rate under 100 beats per minute.20 Each experimental subject performed eight stretches for two sets of 30 seconds.^21^,^22^

Subjects in the AerG exercised three times weekly for 45 minutes at an intensity of 60% of their individual age-predicted maximum heart rate (HR_max_) using a combination of treadmills, rowers, steppers and cycle ergometers. Age-predicted maximum heart rates were determined by subtracting their age from 220. Aerobic exercise intensity was readjusted every four weeks with a 5% increase in heart rate, and the exercise programme was adjusted accordingly.

Subjects in the ResG performed three sets of 15 repetitions for shoulder press, latissimus dorsi pull-downs, seated rows, unilateral leg presses, unilateral knee extensions and unilateral hamstring curls at 60% of the estimated 1-RM^23^ during each training session, using a combination of Polaris® weight machines and York® free weights. The subjects were also required to perform three sets of crunches at 60% of the maximum number of repetitions performed in one minute during the baseline testing.^24^ A 60- to 90-second rest period was observed between each set and each of the various exercises.^25^ Every four weeks, each subject’s estimated 1-RM was re-evaluated and his exercise programme was adjusted accordingly.^24^

In an attempt to equalise for time across the three exercising groups, the present investigation made use of sessions that utilised both aerobic and resistance training in equal durations. Therefore, each subject in the ConG used resistance training for 22 minutes, equating to two sets of 15 repetitions at a workload of 60% 1-RM, or in the case of crunches, the maximum number of repetitions performed in one minute during the baseline testing. As with the ResG, every four weeks each subject’s estimated 1-RM was re-evaluated and his exercise programme adjusted accordingly. The ConG subjects were also required to exercise using a combination of treadmills, rowers, steppers and cycle ergometers for 22 minutes at an intensity of 60% of their individual age-predicted heart rate maximum. This intensity was re-adjusted every four weeks by a 5% increase in heart rate.

While subjects in the exercise groups were not permitted to engage in any form of exercise other than their prescribed intervention programmes, the NonG subjects were not allowed to take part in any structured exercise and had to continue with their inactive lifestyles.

## Statistics

Data were analysed using commercial software [Statistical Package for Social Sciences (SPSS) Version 11, Chicago, IL]. Standard statistical methods were used for the calculation of the homogeneity, means and standard deviation (SD). The differences in VO_2max_ were compared using an analysis of variance (ANOVA) with a Bonferroni *post-hoc* test. Data were presented as means ± standard deviations and the alpha level was set at 0.05.

## Results

Mean values for VO_2max_ for the pre- and post-test are shown in Table 2. The results indicated that the four groups were homogenous at pre-test (*p* = 0.361). While the NonG were found to have decreased their VO_2max_ by a mean of 0.92 ml.kg^-1^.min^-1^ (3.36%), the three exercise training groups all increased their mean VO_2max_. In the ResG, VO_2max_ increased by 4.69 ml.kg^-1^.min^-1^, or an effective 13.16%, whereas the AerG and ConG were found to have increased their VO_2max_ by 10.18 ml.kg^-1^.min^-1^ (34.12%) and 7.93 ml.kg^-1^.min^-1^ (29.58%), respectively. Post-hoc analysis revealed that aerobic training was more effective than no exercise (*p* = 0.000) and resistance training (*p* = 0.041), but was as effective as concurrent training (*p* = 0.365).

**Table 2:** Training Effects On VO_2MAX_ Data Following 16 Weeks Of Aerobic Training, Resistance Training And Concurrent Aerobic And Resistance Training

*Group*	*Mean pre-test (ml.kg^-1^.min^-1^)*	*Mean post-test (ml.kg^-1^.min^-1^)*
AerG	29.81 ± 4.83	39.98 ± 4.87
ResG	35.71 ± 4.39	40.39 ± 6.27
ConG	26.81 ± 6.62	34.74 ± 5.92
NonG	27.41 ± 3.59	26.49 ± 4.55

Values are means ± standard deviation.

The mean body mass of both the AerG and ResG decreased significantly from the pre- to post-test (*p* = 0.008 and *p* = 0.041, respectively), whereas the body mass of the subjects in the ConG and NonG was unchanged (*p* = 0.507 and *p* = 0.695, respectively). Percentage body fat decreased following 16 weeks of aerobic, resistance and concurrent training (*p* = 0.002, *p* = 0.009 and *p* = 0.002, respectively), but not following 16 weeks of no exercise (*p* = 1.000).

## Discussion

Development of aerobic capacity was clearly evident following 16 weeks of aerobic training, resistance training and concurrent training. However, the significant reductions in body mass and percentage body fat observed following the exercise training programmes could explain the concomitant increases in VO_2max_. A possible reason for the smaller changes observed in VO_2max_ following resistance training could be related to the exercise dose, workload, intensity and repetitions of the programme.

From the results of the study, it appears that the interference effect may only hold true in specific situations, and may be related to differences in design factors, including the mode, frequency, duration and intensity of training, the training history of participants, the scheduling of training sessions, selection of dependent variables, age of participant, the upper limit of the subject’s genetic potential and the way in which the two modalities of exercise were integrated. This is because the results suggest that increases in VO_2max_ are not impeded by combining resistance and aerobic training when compared with aerobic training alone in a sample of previously sedentary or untrained, apparently healthy males.

Therefore, the present study supports the concurrent use of resistance and aerobic training in the prevention of cardiovascular disease, since this mode of training may not only increase VO_2max_, but also allow an individual to elicit the unique benefits of each mode of exercise.
